# Comparison of Standard and Point-of-Care CD4+ T Lymphocyte Measurement Methods in HIV-1 Infected Turkish Patients

**DOI:** 10.3390/medicina60122094

**Published:** 2024-12-21

**Authors:** Müge Toygar Deniz, Sıla Akhan, Murat Sayan, Sibel Balcı

**Affiliations:** 1 Department of Infectious Diseases and Clinical Microbiology, Faculty of Medicine, Kocaeli University, Kocaeli 41001, Turkey; cetinakhan@yahoo.com.tr; 2 PCR Unit, Clinical Laboratory, Faculty of Medicine, Kocaeli University, Kocaeli 41001, Turkey; sayanmurat@hotmail.com; 3 DESAM Institute, Near East University, Nicosia 99138, Cyprus; 4 Department of Biostatistics and Medical Informatics, Faculty of Medicine, Kocaeli University, Kocaeli 41001, Turkey

**Keywords:** CD4-Positive T-Lymphocytes, flow cytometry, HIV, point-of-care testing

## Abstract

*Background and Objectives*: CD4+ T lymphocytes are the primary targets of HIV infection. CD4+ T lymphocyte count is an indicator of immune competence. In this study, we aimed to compare standard flow cytometry and point-of-care (POC) CD4+ T lymphocyte in terms of cost, effectiveness, reliability, time, and the use of this method for disease. *Materials and Methods*: This study includes 113 patients. CD4+ T lymphocyte count and percentage were evaluated by flow cytometry and POC. Also, hemoglobin (Hb) level was studied. The data obtained by two methods are compared. *Results*: When the two methods were compared, intraclass coefficients demonstrated a good consistency for Hb (ICC = 0.849) and CD4+ T lymphocyte percentage (ICC = 0.803). For CD4+ T lymphocyte count, consistency was moderate, ICC = 0.651, but still statistically significant (*p* < 0.001). *Conclusions*: In resource-limited countries, virological monitoring with HIV RNA cannot be performed at any time because it is expensive. However, CD4+ T lymphocyte count and percentage monitoring is important in predicting treatment success. POC results are in good consistency with the standard method, and it is also a test that can be used due to being cheap, easy, and quick.

## 1. Introduction

HIV-1 infection continues to affect global human health, with an estimated 39.9 million individuals living with HIV-1 and 30.7 million accessing antiretroviral therapy (ART) according to the most recent reports from the Joint United Nations Programme on HIV/AIDS (UNAIDS) [1]. In Turkey, HIV/AIDS testing has been available since 1985, and as of November 2023, there have been 39,737 cumulative confirmed cases of HIV-positive individuals, with 2295 of these cases progressing to AIDS. In Turkey, there is an increasing trend in HIV cases despite global decline [2].

The main target of the HIV infection is CD4+ T lymphocytes. Monocytes, tissue macrophages, natural killer cells, dendritic cells, hematopoietic stromal cells, and microglial cells can be infected with HIV because they contain CD4 antigen [3,4]. The study of 276 untreated individuals living with HIV revealed that the average CD4+ T lymphocyte count decreased by 58 cells/μL per year [5]. This decrease may lead to the development of opportunistic infections and malignancies characteristic of AIDS [6]. Absolute CD4+ T lymphocyte count is a crucial indicator of immune competence and is used to evaluate the stage of the disease, detect delayed diagnoses, determine the need for prophylaxis against opportunistic infections, and assess treatment success. A normal CD4+ T lymphocyte count ranges from 400 to 1600 cells/mm^3^, and a count below 350 cells/mm^3^ suggests a delayed diagnosis [7]. A count below 200 cells/mm^3^ indicates immunosuppression and an increased risk of opportunistic infections [8].

The current standard of care for individuals with HIV is combination antiretroviral therapy (c-ART), which simultaneously targets multiple viral proteins. Patient response to treatment is monitored through regular virological tests, measuring HIV RNA levels, and immunological assessments, which include CD4+ T lymphocyte counts [9]. It is still very difficult to obtain HIV RNA and CD4+ T cell counts in rural health institutions in resource-limited settings [10]. Flow cytometry is the most common method for measuring the CD4+ T lymphocyte count. However, this approach has limitations, as the result is calculated relative to the total number of lymphocytes in the complete blood count and may change in the presence of intervening infections [11]. CD4+ T lymphocyte counts are determined in laboratories where fresh EDTA blood taken from hospitals during the day is transferred and collected; the results can be obtained in an average of 2–14 days or over a longer period, according to the capacity of the laboratory [12]. A rapid and accurate CD4+ T lymphocyte count may be required to rapidly begin treatment, determine the status of the infection, and prevent potential opportunistic infections during a single hospital visit [13].

In this study, we sought to compare standard flow cytometry and point-of-care (POC) CD4+ T lymphocyte measurement analyses with respect to cost, effectiveness, compliance, reliability, time, and the utilization of this method for disease.

## 2. Materials and Methods

This study included 113 people living with HIV (PLWH). The HIV treatment administered to these participants was consistent with the current recommendations provided by the Turkish Republic Ministry of Health and EACS guidelines. The study cohort comprised adult individuals, 18 years of age or older, who were undergoing antiretroviral therapy (ART).

CD4+ T lymphocytes were evaluated utilizing the standard FACS count reference method (Becton Dickinson, San Jose, CA, USA). This method was predominantly conducted via flow cytometry, wherein cells or particles in a suspension were analyzed by enumerating signals as they passed through a chamber illuminated by a laser source. The point-of-care (POC) measurement method developed for rapid CD4+ T lymphocyte quantification employed in our study utilized a Cartridge (BD FACSPresto, San Jose, CA, USA) kit that used fluorochrome-conjugated antibody reagents and integrated reagent quality control. It consisted of fluorescence imaging and absorbance-reading technology with embedded software to analyze patient samples from a single-use disposable cartridge. The indicators available from BD FACSPresto are CD4+ T lymphocyte count and percentage and hemoglobin level, and it is a portable device that can give results in 25 min. In addition, blood was collected in an EDTA tube to determine the hemoglobin level. The demographic information of the patients was obtained from the hospital data system.

### 2.1. Statistical Analysis

All statistical analyses were performed using IBM SPSS for Windows, version 29.0 (IBM Corp., Armonk, NY, USA), and MedCalc 14 (MedCalc Software, Ostend, Belgium). The Kolmogorov–Smirnov test was used to assess the normality assumption. Normally distributed continuous variables were presented with mean ± standard deviation (SD), non-normally distributed continuous variables were presented with median and interquartile range (IQR). Categorical variables were presented with the number of observations and percentages. Dependent group comparisons were carried out with the Wilcoxon signed rank test. Consistency was determined by intraclass correlation coefficient (ICC) and Bland–Altman method. A *p*-value < 0.05 was considered statistically significant.

### 2.2. Ethical Approval

Ethics approval was obtained from the Ethics Committee of Derince Training and Research Hospital with the number 2021/64. Our study is retrospective and the information in the hospital data system that was used was anonymized. All participants were informed about the latest method for measuring CD4+ T lymphocytes. The consent forms utilized in the study were prepared in accordance with the Helsinki declaration, and the participants were accordingly informed of their rights.

## 3. Results

In our study, a total of 113 PLWH were enrolled for treatment. Demographic, laboratory, and medical findings of the patients are shown in Table 1. Among these patients, males (*n* = 96, 85%) predominated over females (*n* = 17, 15%). The age of the patients ranged between 21 and 74. The mean age of the patients was calculated as 43 ± 13 years.

The HIV-1 RNA values before starting treatment were not available for 12 patients. In addition, the CD4+ T lymphocyte results of 80 patients were available in the data system. Before starting treatment, the average CD4+ T lymphocyte was 435 cells/µL and ranged between 15 and 1842. There were eight patients with CD4 counts below 100 cells/µL (10%) and 25 patients with CD4 counts below 350 cells/µL (delayed admission) before treatment (31%).

Twenty-five patients (22%) reported that their symptoms prompted them to seek medical attention at the hospital, while 14 patients (12%) received their diagnosis during a routine medical examination. Seven patients (6%) were diagnosed through pre-intervention screening tests, and two patients (2%) were diagnosed during screening tests prior to blood donation. Additionally, ten patients (8%) were tested due to their partners’ diagnoses, and one individual reported contracting HIV through a tattoo. Symptoms reported upon admission included diarrhea, dysuria, lymphadenopathy, weight loss, perianal abscess, and Kaposi sarcoma. Nine patients presented with an opportunistic infection at the time of diagnosis. These were pulmonary tuberculosis, progressive multifocal leukoencephalopathy (PML), oral candidiasis, toxoplasma brain abscess, pneumocystis jiroveci pneumonia (PCP), cytomegalovirus (CMV) colitis, mycobacterium avium complex (MAC) infection, Kaposi sarcoma, and Hodgkin lymphoma. CMV colitis and PCP were observed concurrently in one patient. MAC and PML were also observed in one patient who subsequently died. Eleven patients were hospitalized and initiated treatment; others continued their follow-up regularly.

**Figure 1:**
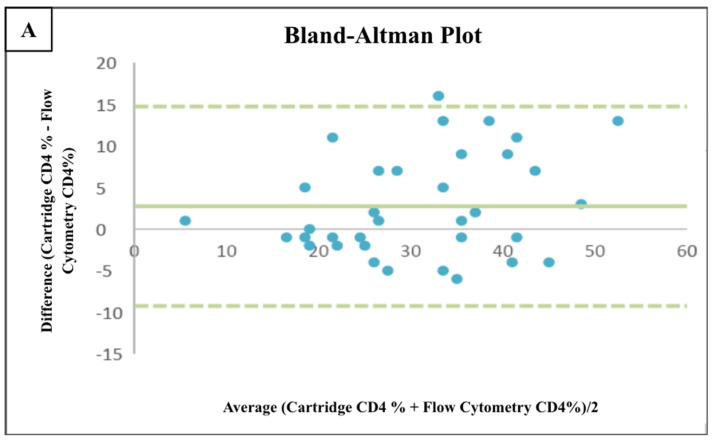

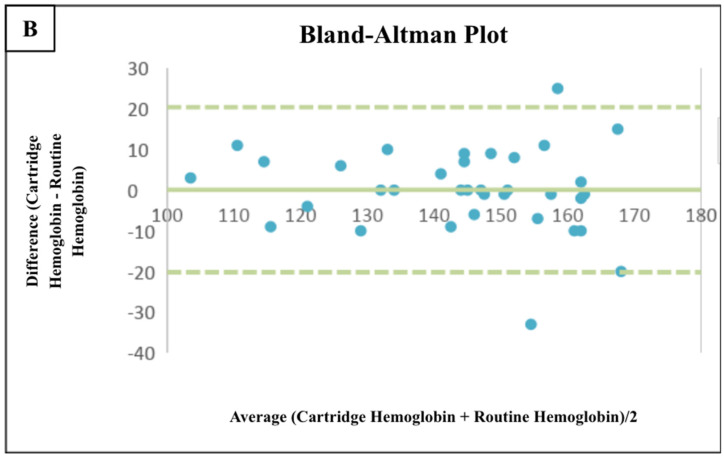
(**A**) Bland−Altman plot comparing the flow cytometry and cartridge CD4 percentage measurements. (**B**) Bland–Altman plot comparing the flow cytometry and cartridge hemoglobin measurements. The center line indicates the mean of differences and the upper and lower horizontal lines at 1.96 times the standard deviations (95% of the data).

In our study, the new CD4+ T lymphocyte count measurement method, called BD FACSPresto (Cartridge), was also used as an analyzer. The key indicators were CD4+ T lymphocyte, percentage, and hemoglobin (Hb) level, and these indicators were obtained via patients’ fingertip blood or plasma sample. The consistency between the results obtained by the Cartridge method and the flow cytometry were evaluated by the Wilcoxon signed rank test and intraclass correlation coefficient (ICC). No statistically significant differences were found between the two techniques with respect to the CD4+ T lymphocyte count, CD4+ T lymphocyte percentage, and Hb levels (*p* = 0.093, *p* = 0.334, *p* = 0.481, respectively) (Table 2). A good consistency was observed between two techniques for CD4+ T lymphocyte percentage (ICC = 0.803, *p* < 0.001) and Hb levels (ICC = 0.849, *p* < 0.001) while a moderate consistency was found for the CD4+ T lymphocyte count (ICC= 0.651, *p* < 0.001) (Table 3). Additionally, a Bland–Altman analysis was performed for CD4+ T lymphocyte percentage and Hb levels since they had a high intraclass correlation coefficient. In the Bland–Altman plots, it was seen that the differences were randomly distributed without any apparent trend and most of the data points fell within the limits of agreement (Figure 1). Mean differences for CD4+ T lymphocyte percentage and Hb levels were not significantly different from zero (*p* = 0.08, *p* = 0.82, respectively) (Table 4). Therefore, no significant bias was observed, and two techniques can be used interchangeably for these parameters.

## 4. Discussion

Today, antiretroviral therapy (ART) has been initiated for every patient diagnosed with chronic HIV infection, regardless of their CD4+ T lymphocyte count [11]. Sub-Saharan African countries are grappling with the difficult decision of when and how to implement the treatments recommended by the guidelines because of limited resources. The BD FACSPresto method is particularly valuable in this context as it facilitates the detection of anemia, which is an adverse effect associated with Zidovudine, a commonly used in resource-limited regions, and allows for CD4+ T lymphocyte monitoring within a single hospital visit. Assessing the immune status of patients through point-of-care (POC) CD4+ T lymphocyte counts is essential for identifying individuals with advanced HIV disease, who should be prioritized for prophylaxis against opportunistic infections, even if they are asymptomatic. Consequently, the measurement of CD4+ T lymphocytes remains a critical component for the timely identification of patients necessitating urgent treatment initiation.

In our study, the POC CD4 measurement method called BD FACSPresto was used. The key indicators of this method were CD4+ T lymphocyte count, CD4+ T lymphocyte percentage, and hemoglobin level [14]. BD FACSPresto provided several significant advantages. Given that the equipment weighs only 7 kg, it is highly portable, allowing patients to obtain their results in just 25 min. Moreover, the availability of various printer options, limited blood volume requirements, and ease of use, as well as the elimination of the need for initial blood processing and preparation of reagents, are all features that would enhance patient compliance. A recent report from Africa has demonstrated that point-of-care CD4 testing can effectively reduce pretreatment loss to follow-up [10]. Similarly, POC tests have been shown to allow access to more people in resource-limited areas [15]. The implementation of POC CD4+ testing technologies in South African healthcare facilities significantly reduced turnaround times for test results and patient loss to follow-up, ultimately improving patient access to antiretroviral therapy [16].

The CD4+ T lymphocyte count was measured by the gold standard (flow cytometry) and was then compared to the BD FACSPresto method, it was seen that the two methods were well correlated. Numerous studies showed the accurate, reliable CD4+ T-lymphocyte and Hb results of this new POC system when compared to the reference method [17,18]. In a study conducted with 300 PLWH in Nigeria, using a cutoff threshold of 500 cells/µL, the sensitivity and specificity of the BD FACSPresto were found to be 95.1% and 97.1%, respectively, when compared to BD FACSCount, which is another method for POC measurement [19]. In the pediatric age group (individuals under 5 years of age), the CD4+ T lymphocyte percentage is considered a more reliable surrogate marker for the immunological status of patients diagnosed with chronic HIV infection [20]. As seen in Table 3, the fact that this parameter had good consistency between both methods in our study may be another reason for the use of the new method.

The BD FACSPresto system generates results that are frequently utilized for monitoring co-infections, opportunistic infections, or treatment failure among HIV-infected populations, particularly where access to viral load testing is lacking. Establishing a baseline CD4 cell count at the start of antiretroviral therapy (ART) continues to provide valuable insight into the immune system’s status for monitoring and long-term care management. Evidence suggests that early initiation of ART has been shown to reduce the hazard ratio for serious AIDS-related events [21]. Similarly, a study by Sagnia et al. showed that BD FACSPresto CD4+ T-lymphocyte count measurement was consistent with the reference method and remains very important in resource-limited settings, where viral load testing is not always available because it is expensive [22].

Furthermore, the hemoglobin test holds significant importance in detecting drug toxicity resulting from zidovudine, a medication commonly utilized in resource-constrained settings. Additionally, Hurst et al. suggested that it could prove to be particularly beneficial in preventing mother-to-child transmission (PMTCT) programs [23]. In our opinion, hemoglobin levels represent the most reliable parameter for comparison between the two methods, as they are determined in a more standardized manner. Consequently, the good consistency of hemoglobin values in our study underscores the reliability of the new method.

Our analysis using the Bland–Altman method revealed a good consistency between the hemoglobin levels measured by the two methods, similar to the findings of a previous study in Uganda [24]. However, this consistency was not observed for the CD4 measurements. For the CD4+ T lymphocyte count, there is a greater error in higher values, with the flow cytometry data tending to report higher levels of CD+ T lymphocytes than expected; however, the difference does not exceed 12%. Therefore, the most reliable parameter to assess the reliability of the new method is the measurement of hemoglobin, since it shows a good consistency with ICC = 0.849 (*p* < 0.001) and the Bland–Altman analysis in Figure 1.

### Study’s Limitations

The limitation of our study is that it was not controlled with an HIV-negative patient group.

## 5. Conclusions

In conclusion, as we found in our study, the high correlation of hemoglobin values may give an idea about the reliability of the new method and POC technology is a cost-effective alternative that increases patient compliance, especially in resource-limited regions. The BD FACSPresto system is a method that can be used because it is cheap, easy, and gives quick results.

## Figures and Tables

**Table 1:** Demographic, laboratory, and medical findings of the patients.

Characteristic	Patient Group (*n* = 113)
Gender; M/F, *n* (%)	96 (85)/17 (15)
Age (years), mean + SD	43 ± 13
Basal HIV-1 RNA load × 10^4^ (IU/mL), median (IQR)	30.9 (5.24–130.5)
Basal Standart CD4+ T lymphocyte count (cells/µL), median (IQR)	649 (441.5–889)
Diagnostic status in CD4+ T lymphocyte count (cells/µL), *n* (%)	
CD4 ≤ 100	8 (10)
100 < CD4 ≤ 350	25 (31)
350 < CD4 ≤ 500	18 (22)
CD4 > 500	29 (36)
Treatment status, *n* (%)	
ABC + DTG + 3TC	5 (4)
TDF/FTC/c/EVG	2 (2)
TAF/FTC/c/EVG	57 (50)
TDF/FTC + DTG	14 (12)
TAF/FTC/BIC	33 (30)
TDF/FTC + LPV/r	1 (1)
RAL + ZDV + TDF	1 (1)

Abbreviations: M—male, F—female, CD—cluster of differentiation, ABC—abacavir, DTG—dolutegravir, 3TC—lamivudine, EVG—elvitegravir, c—cobicistat, TDF—tenofovir disoproxil fumarate, FTC—emtricitabine, TAF—tenofovir alafenamide, BIC—bictegravir, LPV—lopinavir, r—ritonavir, RAL—raltegravir, ZDV—zidovudine.

**Table 2:** Comparisons of flow cytometry and cartridge techniques.

	Flow Cytometry	Cartridge	*p* *
CD4+ T lymphocyte count (mm^3^/µL), median (IQR)	649 (441.5–889)	685 (500.5–949)	0.093
CD4+ T lymphocyte percentage (%), median (IQR)	29 (23–39.5)	30 (24.5–36)	0.334
Hemoglobin (mg/dL), median (IQR)	150 (139.5–157.5)	149 (139–161.5)	0.481

* Wilcoxon signed rank test. Abbreviations: IQR—interquartile range.

**Table 3:** Consistency of flow cytometry and cartridge techniques.

	ICC	*p*
CD4+ T lymphocyte count (mm^3^/µL)	0.651	<0.001
CD4+ T lymphocyte percentage (%)	0.803	<0.001
Hemoglobin (mg/dL)	0.849	<0.001

Abbreviations: ICC—Intraclass correlation coefficient.

**Table 4:** Bland–Altman analysis results in study patients.

Parameter	CD4+ T Lymphocyte Count (mm^3^/µL)	CD4+ T Lymphocyte Percentage (%)	Hemoglobin (mg/d)
Mean Difference	54	−1.4	0.25
SD	329.65	8.9	12.6
95% CI	−7.07 to 115.81	−3.15 to 0.18	−2.10 to 2.61
*p*	0.08	0.08	0.82
Aggrement Limits			
Lower limit	−591.74	−19.01	−24.55
95% CI	−697.06 to −486.42	−21.87 to −16.15	−28.60 to −20.51
Upper limit	700.49	16.05	25.07
95% CI	595.17 to 805.80	13.19 to 18.90	21.02 to 29.11

Abbreviations: CD—Cluster of differentiation, SD—Standard deviation, CI—Confidence interval.

## Data Availability

The original contributions presented in the study are included in the article, further inquiries can be directed to the corresponding author.
